# Synovectomy during total knee arthroplasty: a pilot single-centre randomised controlled trial

**DOI:** 10.1186/s40814-018-0336-y

**Published:** 2018-08-25

**Authors:** Kenneth S. Rankin, Jayasree Ramaskandhan, Michelle Bardgett, Katie Merrie, Rajkumar Gangadharan, Ian Wilson, David Deehan

**Affiliations:** 1 Musculoskeletal Department, Freeman Hospital, High Heaton, Newcastle upon Tyne, NE7 7DN UK; 2 Institute of Genetic Medicine, University of Newcastle upon Tyne, International Centre for Life, Central Parkway, Newcastle upon Tyne, NE1 3BZ UK

**Keywords:** Knee, Osteoarthritis, Synovectomy

## Abstract

**Background:**

Total knee arthroplasty (TKA) is an effective procedure for late-stage osteoarthritis (OA) of the knee; however, up to 20% of patients remain dissatisfied. In some patients, this may be due to residual inflammation of the synovium. Our aim was to perform the first randomised controlled trial (RCT) of synovectomy during TKA for patients with macroscopically inflamed synovium. The main objectives were to assess recruitment rates, protocol adherence and outcomes relating to safety such as haemoglobin decrease and adverse events. We also collected data on patient-reported outcomes.

**Methods:**

We performed a single-centre pilot RCT. Patients with a macroscopically inflamed synovium were randomised to receive synovectomy versus a control group that did not undergo synovectomy. We determined feasibility by measuring patient enrolment, completeness of follow-up, and safety via haemoglobin decrease and documentation of adverse events.

**Results:**

We screened 360 patients with 260 deemed ineligible or could not be recruited. From the 100 eligible patients, 54 were enrolled and 40 progressed through to randomisation. All made it to the 12-month follow-up, indicating good protocol adherence. There were no major differences in adverse events or haemoglobin decrease demonstrating acceptable safety. Outcomes relating to satisfaction were reliably obtained.

**Conclusions:**

Patients with macroscopically inflamed synovium of the knee who are due to undergo TKA can be reliably recruited to a randomised trial and synovectomy can be performed safely. A large number is needed to be screened to identify eligible participants, and therefore, a multi-centre trial would be required to assess whether routine synovectomy would improve outcomes in these patients.

**Trial registration:**

ISRCTN, ISRCTN31010214. Registered 6 October 2016—retrospectively registered

**Electronic supplementary material:**

The online version of this article (10.1186/s40814-018-0336-y) contains supplementary material, which is available to authorized users.

## Background

Osteoarthritis (OA) is the most common disease affecting synovial joints in the middle- to old-age population [1]. The knee is one of the most frequent anatomical locations involved, which results in a massive health economic burden related to physical disability [2]. Late-stage osteoarthritis of the knee is defined as severe pain not controlled with regular analgesic medication accompanied by radiographic evidence of joint space narrowing, osteophyte formation and subchondral sclerosis. Due to a complete lack of disease-modifying osteoarthritis agents, surgery in the form of total knee arthroplasty (TKA) remains the most effective method of managing late-stage cases [3]. There are an increasing number of patients receiving TKA which is due to improving knee implant designs and a lowering of thresholds for surgeons to offer surgery [4]. In the UK, over 85,000 patients undergo TKA each year [5], and in the USA, this figure is over 620,000 [6]. With the economic impact of the projected 56,918 patients requiring revision surgery which is due to reach $2 billion annually by 2030, any reduction in the requirement for further procedures would be welcome [7].

Survival for most implant designs with revision taken as an endpoint is over 95% at 10 years; however, satisfaction analyses indicate consistently that at least 20% of patients are dissatisfied with their knee replacement [8]. The variability in macroscopic appearance of the synovium has been noted by surgeons performing TKA [9], and the historical description of OA as a non-inflammatory disease has been superseded by clinical [10], radiological [11–14], and pathological [15] evidence of inflammatory processes, particularly in the knee joint. There is concern, therefore, that a proportion of patients reporting unsatisfactory outcomes following TKA may have persistent inflammatory activity in the knee joint driven by the presence of residual synovitis [16]. There have been three surgical trials assessing synovectomy during TKA. All concluded there is no benefit to performing synovectomy; however, in all of these studies, there was no intra-operative attempt to discriminate between inflamed and non-inflamed synovium [17–19].

Our aim was to perform the first randomised controlled trial (RCT) of synovectomy versus no synovectomy during TKA for OA in patients with macroscopically inflamed synovium. The main objectives were to assess recruitment rates, protocol adherence, and outcomes relating to safety such as haemoglobin decrease and adverse events. We also collected data on patient-reported outcomes.

## Methods

### Study design and setting

Ethical approval was obtained (Regional Ethics Committee reference: 10/H0904/76) followed by permission from our local Research Governance Department. The study set up commenced in June 2013, and final follow-up was in October 2015. Participants were recruited between 1 November, 2013, and 7 October, 2014. The study was performed at a university hospital in the UK. Forty patients who are due to undergo a routine primary total knee arthroplasty (TKA) procedure under the care of three senior surgeons completed participation in the study. The study is registered to the ISRCTN: 31010214. The schedule of the trial (Additional file 1) is available as supplementary material.

### Participants/study subjects

Inclusion criteria were patients over the age of 18 years with a diagnosis of late-stage knee osteoarthritis. Exclusion criteria were as follows: refusal or inability to provide informed consent; those unable to answer questionnaires for cognitive reasons; patients diagnosed with auto-immune inflammatory arthritis of the knee; morbidly obese individuals (with BMI > 40); patients with a neuromuscular disorder that would affect their ability to mobilise; patients undergoing bilateral synchronous knee replacement procedures; patients undergoing more complex surgery requiring an implant with increased constraint including posterior cruciate sacrificing designs. Written consent was obtained from those agreeing to participate.

### Randomisation and blinding

**Fig. 1:**
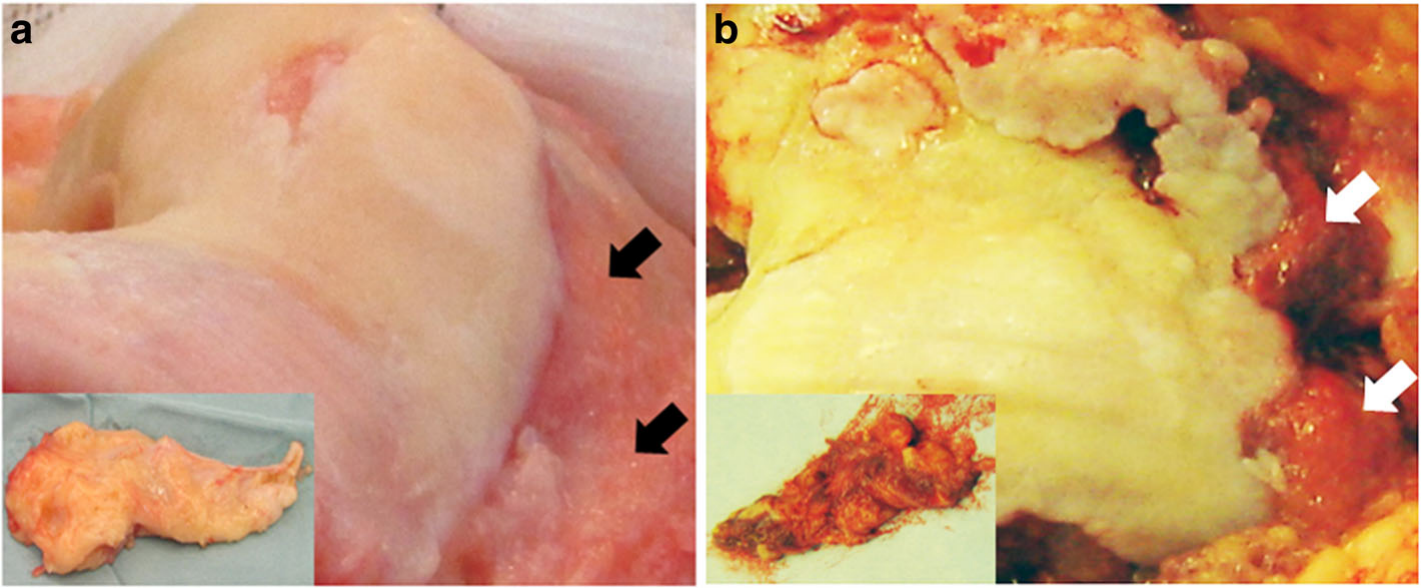
Representative photographs of non-inflamed versus inflamed synovium. **a** Synovium that is not macroscopically inflamed. Black arrows: on the knee. Inset: small representative specimen. Patient is withdrawn from the study. **b** Synovium that is macroscopically inflamed. White arrows: on the knee. Inset: small representative specimen. Patient is randomised intra-operatively

The trial consisted of two arms, and randomisation was performed intra-operatively by a research nurse attending the operating room. After eversion of the patella, the synovium was evaluated by the surgeon. Evidence of hyperplasia, papilla formation and engorgement was defined as being consistent with a macroscopically inflamed synovium and deemed an inclusion criterion (Fig. 1). Patients who did not have a macroscopically inflamed synovium underwent a standard surgical procedure and were withdrawn from the study. Participants with confirmed macroscopically inflamed synovium were randomised to receive synovectomy or no synovectomy and underwent a standard implantation of the prosthesis. Participants were blinded until their 1-year follow-up. Block randomisation was performed by a research nurse using the ‘sealed envelope.com’ website to provide the allocation until 20 participants had been recruited into each arm. The research nurse was not involved in any further aspects of the trial.

### Interventions

All operations were performed using a standard surgical approach with prior application of a high thigh tourniquet. The procedure consisted of a midline incision, a medial parapatellar approach and eversion of the patella. Hoffa’s fat pad in all cases was excised to facilitate access to the joint. A cemented cruciate retaining implant was used in all cases. No patients received tranexamic acid. Antibiotic and thromboprophylaxis protocols were identical for both groups, and post-operative rehabilitation was the same. For participants randomised to synovectomy, a meticulous technique was undertaken to avoid significant blood loss. This involved resection of the intimal and subintimal layers, leaving the vascular layer intact.

### Follow-up

Our team of research physiotherapists followed participants at 6 weeks and 1 year to document clinical outcomes and patient-reported outcome measures. In addition, haemoglobin decrease day 1 post-operatively was documented. The clinical outcome measure was improvement in range of movement assessed pre-operatively and at 6 weeks and 1 year post-operatively. The patient-reported outcome measures evaluated included disease orientated, satisfaction and general quality of life scores. The Western Ontario and McMaster Universities osteoarthritis index (WOMAC) provides general information about the impact of the disease process on daily life [20] and was measured pre-operatively and 1 year post-operatively. A score for patient-reported co-morbid medical conditions was included to demonstrate that there were no participants with a disease process other than OA that would hinder their rehabilitation [21]. The Short Form 36 health survey (SF-36) [22], and EQ-5D [23] are general quality of life scores which have been demonstrated to improve following successful TKA and were therefore measured pre-operatively and 1 year post-operatively. Finally, a patient satisfaction from surgery score was obtained using a validated outcome measure [24]; this questionnaire includes four questions about satisfaction with overall outcome, pain relief, ability to perform activities of daily living and ability to participate in leisure activities. Responses are on a 4-point Likert scale, which ranges from very satisfied to very dissatisfied. The satisfaction score was measured at 1 year post-operatively. Complications were recorded using a standard proforma. Participants were given a telephone helpline number to contact the study team in the event of any adverse events. In addition, at the 6-week follow-up, the research team actively sought out any possible adverse events from the participants.

### Statistical analyses

A sample size was not calculated due to the design of the study, i.e. pilot trial. The aim was to screen, enrol and randomise as many patients as possible over a 12-month recruitment period and stop recruitment when 20 to 30 patients were enrolled into each arm. Statistical analysis was performed on the clinical range of movement data to assess for improvement from the 6-week to 1-year time points which should be demonstrable in a small TKA group and also to assess for a difference between the intervention and no intervention groups. An analysis was also performed to check for a significant difference in haemoglobin decrease between the intervention and no intervention groups. The analysis was performed using SPSS software version 17 (SPSS Inc., Chicago, IL). The data were checked for normal distribution using the Kolmogorov-Smirnov test. The two sample *t* test was used for comparison of means for the SF-36, WOMAC and EQ-5D scores. Results are presented with 95% confidence intervals, and any hypothesis testing is preliminary and should be interpreted with caution.

## Results

### Recruitment

The participants were recruited between 1 November, 2013, and 7 October, 2014. Three hundred sixty patients were screened. Two hundred sixty were found to be ineligible or could not be recruited. Fifty-four of the eligible patients agreed to participate, and informed consent was obtained. A detailed summary of the attrition from screening is shown in Table 1.

**Table 1:** Attrition from screening to enrolment

360 screened	
196 ineligible	
46 listed for surgery under private healthcare	
37 listed for revision rather than primary TKA	
27 due to undergo bilateral synchronous TKA	
24 not a participating surgeon	
23 auto-immune inflammatory joint disease	
16 BMI > 40	
8 co-existent medical problem	
4 screened before official study start date	
3 inability to provide informed consent	
3 listed for NHS waiting list Saturday surgery	
2 cognitive reasons	
2 not listed for primary TKA	
1 fixed motor deficit	
164 initially eligible but 22 not given study information or approached	
15 recruitment closed	
7 surgery time changed so unable to approach	
142 were sent a patient information sheet and 42 had no further contact or approach	
10 recruitment had closed	
8 surgery cancelled or time changed	
7 surgeon not participating	
7 no staff available to approach	
3 listed for NHS waiting list Saturday surgery	
3 discovered BMI > 40	
2 listing changed to private healthcare	
1 fixed motor deficit	
1 deceased	
100 patients contacted or approached: 21 declined and 25 discovered ineligible	
8 surgeon not participating	
5 surgery cancelled or time changed	
3 listed for bilateral synchronous TKA	
3 discovered BMI > 40	
2 inability to provide informed consent	
1 fixed motor deficit	
1 listed for complex TKA	
1 listing changed to private healthcare	
1 listed for NHS waiting list Saturday surgery	
54 enrolled	
14 withdrawn due to intra-operative finding of non-inflamed synovium	
40 randomised and followed up	

**Fig. 2:**
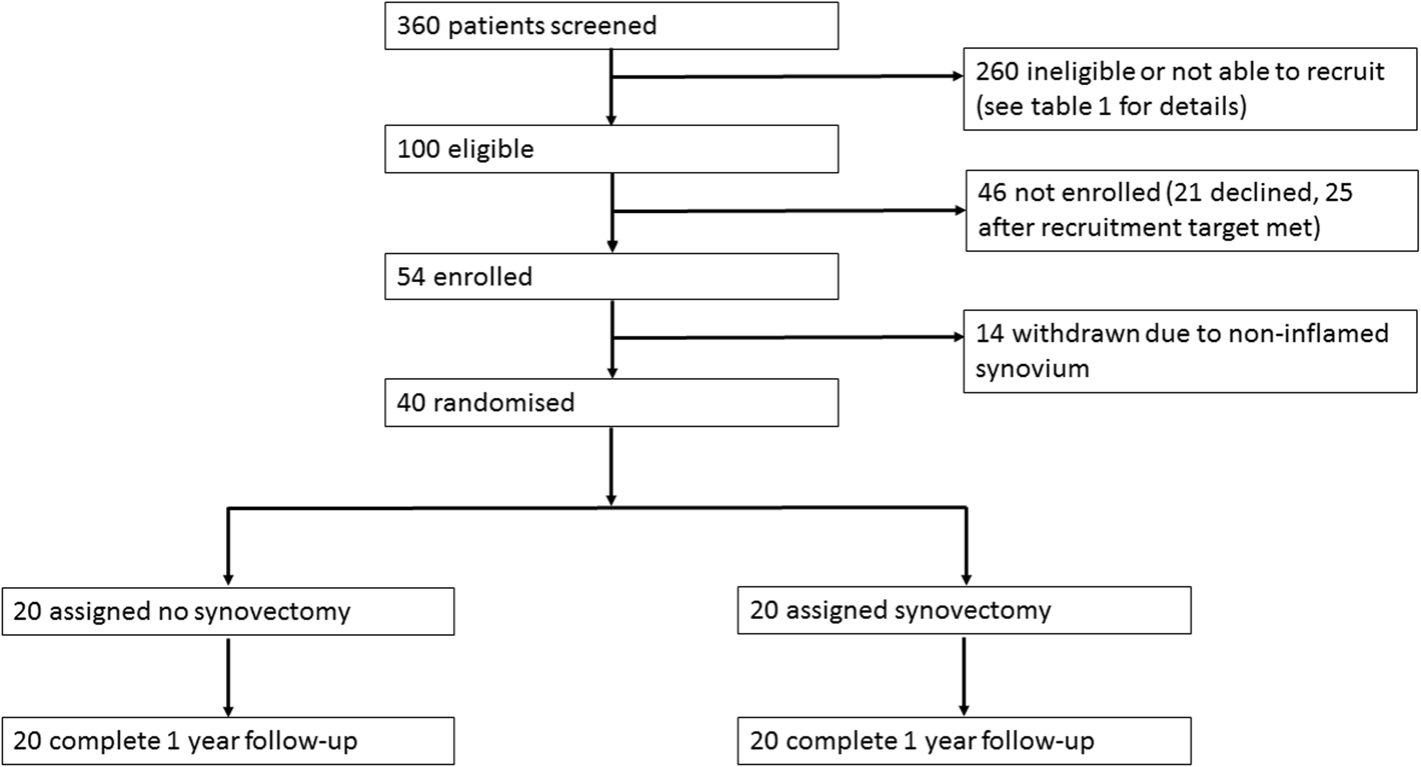
CONSORT flow chart

Fourteen participants were subsequently withdrawn due to the finding of a non-inflamed synovium intra-operatively. Twenty participants were randomised to each group. Of the 40 participants, all were successfully followed up to the 1-year conclusion with no drop outs, as per the CONSORT diagram (Fig. 2).

### Baseline characteristics

There were 40 patients in the study with 20 participants in each arm (Table 2). There were 29 males and 11 females. Mean age was 67.5 for the no synovectomy group and 69.1 for the synovectomy group.

**Table 2:** Baseline characteristics of the participants according to intervention

	No synovectomy (*n* = 20)	Synovectomy (*n* = 20)
Sex		
Male, *n* (%)	13 (65%)	16 (80%)
Female, *n* (%)	7 (35%)	4 (20%)
Age in years (range)	67.5 (44.6–83.3)	69.1 (54.6–85.4)
Average number of co-morbidities mean (SD)	1.4 (1.1)	1.5 (1.4)
Side of operation		
Right, *n* (%)	12 (60%)	7 (35%)
Left, *n* (%)	8 (40%)	13 (65%)
BMI (kg/m^2^) mean (SD)	28.7 (3.2)	30.1 (3.4)

### Safety

There was a decrease in mean haemoglobin levels observed in both groups from pre-operatively to day one post-operatively: no synovectomy 138 g/L (SD 14) to 118 g/L (SD 16) versus synovectomy 141 g/L (SD 11) to 117 g/L (SD 10). There was no difference in mean haemoglobin levels between the two groups both pre-operatively relative risk − 2.90, 95% CI − 10.9 to 5.1, *p* = 0.467, and post-operatively relative risk 0.30, 95% CI − 8.36 to 8.96, *p* = 0.944. No patient received a blood transfusion.

There were 10 adverse events reported during the study (Table 3). Two were serious adverse events due to hospitalisation of the participants, both of whom were in the synovectomy group. In one case, this was due to constipation, and in the second case, the participant had developed a superficial wound infection. This was treated with antibiotics only and did not require surgical intervention. These serious adverse events were not classified as related to the synovectomy procedure.

**Table 3:** All adverse events

	No synovectomy (*n* = 20)	Synovectomy (*n* = 20)
Total adverse events	3	7
Serious adverse events		
Death	0	0
Life threatening	0	0
Admission to hospital	0	1
Constipation		Resolved with laxatives
Admission to hospital	0	1
Superficial wound infection		Resolved with antibiotics
Adverse events		
Suspected DVT (excluded)	1	2
Suspected DVT (confirmed)	0	0
Hip pain	1	0
Nausea	0	1
Wound problem	1	1
Urinary tract infection	0	1

### Feasibility

All 40 randomised patients completed follow-up to the 1-year end point. There was 100% documentation of range of movement scores at all time points.

For the patient-reported outcome measures, completeness of follow-up was 100% for the EQ-5D, 95% for the WOMAC and SF-36 scores and 87.5% for the patient satisfaction from surgery scores (Table 4). The loss of data was due to illegible recording on the scoresheet by the participants. There were no protocol deviations.

**Table 4:** Completeness of follow-up

Outcome	Pre-operative, *n* (%)	6 weeks, *n* (%)	1 year, *n* (%)
Range of movement	40 (100)	40 (100)	40 (100)
WOMAC	38 (95)	n/a	38 (95)
SF-36	38 (95)	n/a	38 (95)
EQ-5D	40 (100)	n/a	40 (100)
Satisfaction score	n/a	n/a	35 (87.5)

*WOMAC* Western Ontario and McMaster Universities Arthritis Index, *SF-36* Short form 36, *EQ-5D* Euroqol Five Dimension Scale

### Secondary outcomes

There was no difference in range of movement within the groups from pre-operatively to 1 year post-operatively (*p* = 0.602). There was a significant improvement in knee range of motion from 6 weeks post-operatively to 1 year post-operatively for both groups (no synovectomy: 100.5° (SD 14.1) to 113.8° (SD 12.6), *p* < 0.0001; synovectomy: 97.5° (SD 12.5) to 114.2° (SD 11.4), *p* < 0.0001). For the EQ-5D Health score, the no synovectomy group scored lower than the synovectomy group at 1 year (81.00 versus 85.63), but this did not achieve statistical significance (Table 5).

**Table 5:** EQ-5D pre-operatively and 1 year post-operatively. Numbers are mean (SD)

	No synovectomy	Synovectomy	95% CI
Pre-operative	*n* = 20 77.55 (17.57)	*n* = 20 77.05 (16.07)	0.50 (− 10.29 to 11.29)
One year post-operative	*n* = 19 81.00 (14.15)	*n* = 16 85.63 (13.26)	− 4.63 (− 14.07 to 4.82)

*EQ-5D* Euroqol five dimension scale, *SD* standard deviation, *CI* confidence interval

There was a significant improvement in mean SF-36 scores from pre-operative to 1 year post-operatively for all domains (*p* < 0.0001) within both groups. There was no statistically significant difference between the groups at the 1-year follow-up (Table 6).

**Table 6:** SF-36 1 year post-operatively. Numbers are mean (SD)

SF-36 domains	No synovectomy	Synovectomy	95% CI
Physical functioning	*n* = 19 60.0 (26.2)	*n* = 18 74.1 (25.2)	− 14.10 (− 31.29 to 3.08)
Role-physical	*n* = 19 64.5 (33.1)	*n* = 19 77.6 (29.0)	− 13.20 (− 33.60 to 7.30)
Bodily pain	*n* = 19 59.2 (30.8)	*n* = 19 72.3 (25.1)	− 13.11 (− 31.62 to 5.41)
General health	*n* = 19 66.3 (21.7)	*n* = 19 61.7 (23.1)	4.55 (− 10.22 to 19.31)
Vitality	*n* = 19 60.2 (18.3)	*n* = 19 64.5 (23.4)	− 4.28 (− 18.12 to 9.57)
Social functioning	*n* = 19 71.1 (24.7)	*n* = 19 84.9 (23.0)	− 13.82 (− 29.54 to 1.91)
Role-emotional	*n* = 19 86.8 (25.8)	*n* = 19 82.0 (30.1)	4.82 (− 13.63 to 23.28)
Mental health	*n* = 19 77.9 (18.5)	*n* = 19 80.9 (17.5)	− 2.96 (− 14.82 to 8.90)

*SF-36* Short Form 36, *SD* standard deviation, *CI* confidence interval

There was a significant improvement in mean WOMAC scores for the no synovectomy group (pain 39.7 (SD 15.3) to 78.6 (SD 21.0); function 40.3 (SD 19.3) to 74.3 (SD 23.3); stiffness 40.1 (SD 21.4) to 76.3 (SD 19.5); *p* < 0.0001) and the synovectomy group (pain 43.6 (SD 15.0) to 83.9 (SD 21.2); function 48.1 (SD 11.6) to 84.6 (SD 16.6); stiffness 50.0 (SD 18.1) to 80.1 (SD 15.3); *p* < 0.0001). There was no significant difference between groups in mean WOMAC scores for pain (*p* = 0.448), function (*p* = 0.131) and stiffness (*p* = 0.531) at 1 year post-operatively (Table 7).

**Table 7:** WOMAC scores 1 year post-operatively. Numbers are mean (SD)

	No synovectomy	Synovectomy	95% CI
Pain	*n* = 19 78.6 (21.0)	*n* = 19 83.9 (21.2)	− 5.26 (− 19.20 to 8.67)
Function	*n* = 19 74.3 (19.3)	*n* = 18 84.6 (16.6)	− 10.29 (− 28.30 to 3.22)
Stiffness	*n* = 18 76.3 (19.5)	*n* = 17 80.1 (15.3)	− 3.76 (− 15.86 to 8.34)

*WOMAC* Western Ontario and McMaster Universities Arthritis Index, *SD* standard deviation, *CI* confidence interval

**Fig. 3:**
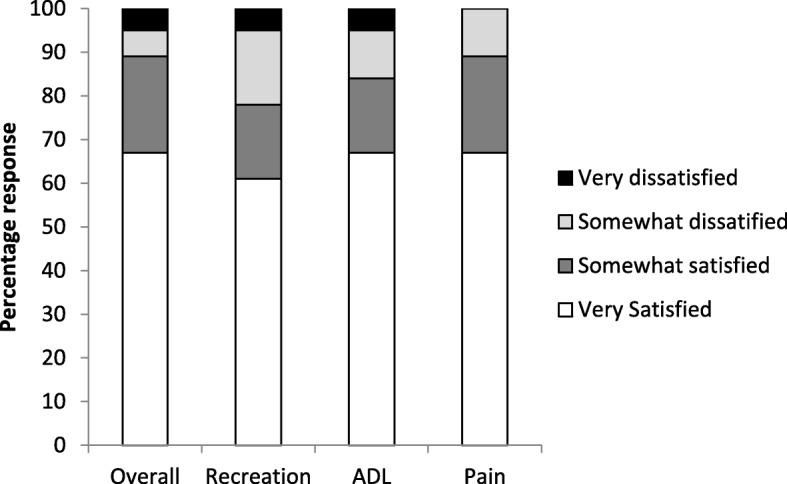
Patient satisfaction at 1 year, no synovectomy (*n* = 18). ADL, activities of daily living

**Fig. 4:**
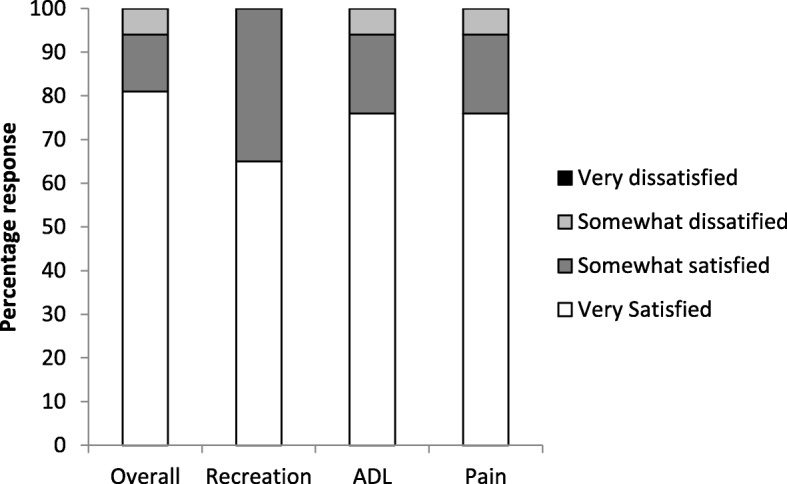
Patient satisfaction at 1 year, synovectomy (*n* = 17). ADL, activities of daily living

There was no difference between groups in terms of patient satisfaction reported for pain relief (synovectomy group 94.1% vs control group 88.9%; proportion difference = 5.2%, 95% CI 63% to 98%), return to activities of daily living (ADL) (synovectomy group 83.4% vs control group 88.5%; proportion difference = 5.1%, 95% CI 60% to 90%), return to recreational activities (synovectomy group 77.8% vs control group 100%; proportion difference = 22.2%, 95% CI 54% to 91%) and overall satisfaction from surgery (synovectomy group 89.9% vs control group 93.8%; proportion difference = 3.9%, 95% CI 67% to 96%) at 1 year post-operatively (Figs. 3 and 4).

## Discussion

The evidence of an inflammatory component of knee OA at the early to late stages of disease is increasing and can even be tracked systemically [25]. At the knee joint, apart from the synovium, there are studies indicating that other tissues such as the fat pad can be a source of inflammation [26]. Furthermore, genomic studies of various joint tissue types from patients with knee OA have implicated epigenetic effects that result in the upregulation of pro-inflammatory cytokines [27] and the link from inflammation to the stimulation of pain pathways in knee OA is well established [28]. From a clinical perspective, a case series of arthroscopic debridement and synovectomy in the moderate to severe stages of knee OA has shown some benefit; however, this was temporary due to the progressive nature of the pathology [29]. Therefore, TKA remains the treatment of choice for late-stage disease but the significant proportion of dissatisfied patients continues to be a challenge. The aim of future therapy for knee OA should include accurate identification of those patients who are at risk of residual pain due to persistent inflammation in the soft tissues following TKA. This would involve assessing clinical, radiological and possibly systemic parameters to define the level of inflammation in the joint prior to embarking on therapy. There have been three randomised controlled trials that have sought to assess the effect of synovectomy during TKA [17–19]. In one of these studies, patients with clinical evidence of synovial thickening were included, but there was no mention of the state of the synovium intra-operatively [18]. The other two studies made no attempt to assess levels of inflammation in the joint [17, 19]. These trials did not discern any benefit from synovectomy, and in two studies, there was evidence of increased blood loss in the intervention group [17, 19]. In one of these trials [17], the participants received bilateral synchronous knee replacements with one side randomised to undergo synovectomy. Blood loss was 107 cc higher in the synovectomy patients, and this did not impact on outcomes. It should be noted that the surgical technique for synovectomy was described in only one of these trials which entailed resection of all the synovial layers, including the vasculature [18]. This is important, because an accurate synovectomy technique that removes the subintimal and intimal layers but leaves the vascular layer intact will result in minimal haemorrhage if the precise tissue planes are identified and respected. In our study, we randomised the participants intra-operatively after confirmation of the macroscopically inflamed synovium and performed a meticulous synovectomy technique retaining the vascular layer. Our trial demonstrates that synovectomy can be performed safely in patients with evidence of synovitis with no detrimental effects. We have also demonstrated that the enrolled participants could be followed up reliably up to 1 year post-operatively with documentation of adverse events and patient-reported outcome measures. In keeping with the difficulties experienced in recruiting to surgical RCTs in general, our study reveals a high rate of attrition from screening to enrolment with a wide range of reasons for eligible patients not making it to the consent stage. This is important because a subsequent trial would need to recruit from multiple centres in order to ensure that the required number of participants to address the potential superiority of the synovectomy intervention would be achieved in a reasonable timeframe. Options for reducing attrition could also be considered such as ensuring that provision is made in subsequent funding applications for the definitive trial for additional staff to screen more effectively and prevent drop out from identification of eligibility to participant approach and enrolment. A further issue was some inconsistency at follow-up. This was mainly due to illegible scores from the patient questionnaires. This could be mitigated by ensuring additional resources are available to request a further score from a participant when the initial submission is not evaluable. A weakness of our study is that the assessment of the synovium was based on a colour judgement by the surgeon with no imaging or tissue sampling to correlate, and therefore, further studies are already underway at our institution to assess whether the synovial tissue judged to be macroscopically inflamed correlates with pre-operative imaging and tissue profiles. A further limitation of our trial is the small sample size which means that all the results should be interpreted with caution. Regarding synovectomy during knee replacement, large multi-centre surgical studies are warranted to assess the potential benefits in larger patient cohorts. Future trial design should include objective measures of inflammation both in the joint tissues and systemically. For those patients that still suffer from pain due to residual inflammation following TKA, adjunctive therapy in the form of biologics that affect relevant cellular pathways is an option to be explored as evidenced by the use of anakinra to improve symptomatology in patients with arthrofibrosis following TKA [30]. For patients with severe pain likely related to synovitis but with minimal changes on the plain radiograph, a combined approach using arthroscopic synovectomy and medical therapies may avoid the requirement for joint replacement. Defining these treatment strategies will require close collaboration between orthopaedic surgeons and rheumatologists in order to design studies which will allow us to develop personalised multi-modal therapeutic for this large group of patients with an awareness that recruiting to these studies will require significant amounts of funding with multi-centre involvement.

## Additional file

Additional file 1: Schedule of the trial. (PPT 259 kb)

## Abbreviations

RCT: Randomised controlled trial
TKA: Total knee arthroplasty
