# Supplementary material for: Synovectomy during total knee arthroplasty: a pilot single-centre randomised controlled trial
Source: Pilot Feasibility Stud. 2018 Aug 25;4:145. doi: 10.1186/s40814-018-0336-y (PMC6109454; doi:10.1186/s40814-018-0336-y)
Supplement: Supplementary file 1 — Schedule of the trial. (PPT 259 kb) [file 40814_2018_336_MOESM1_ESM.ppt]

## Slide 1
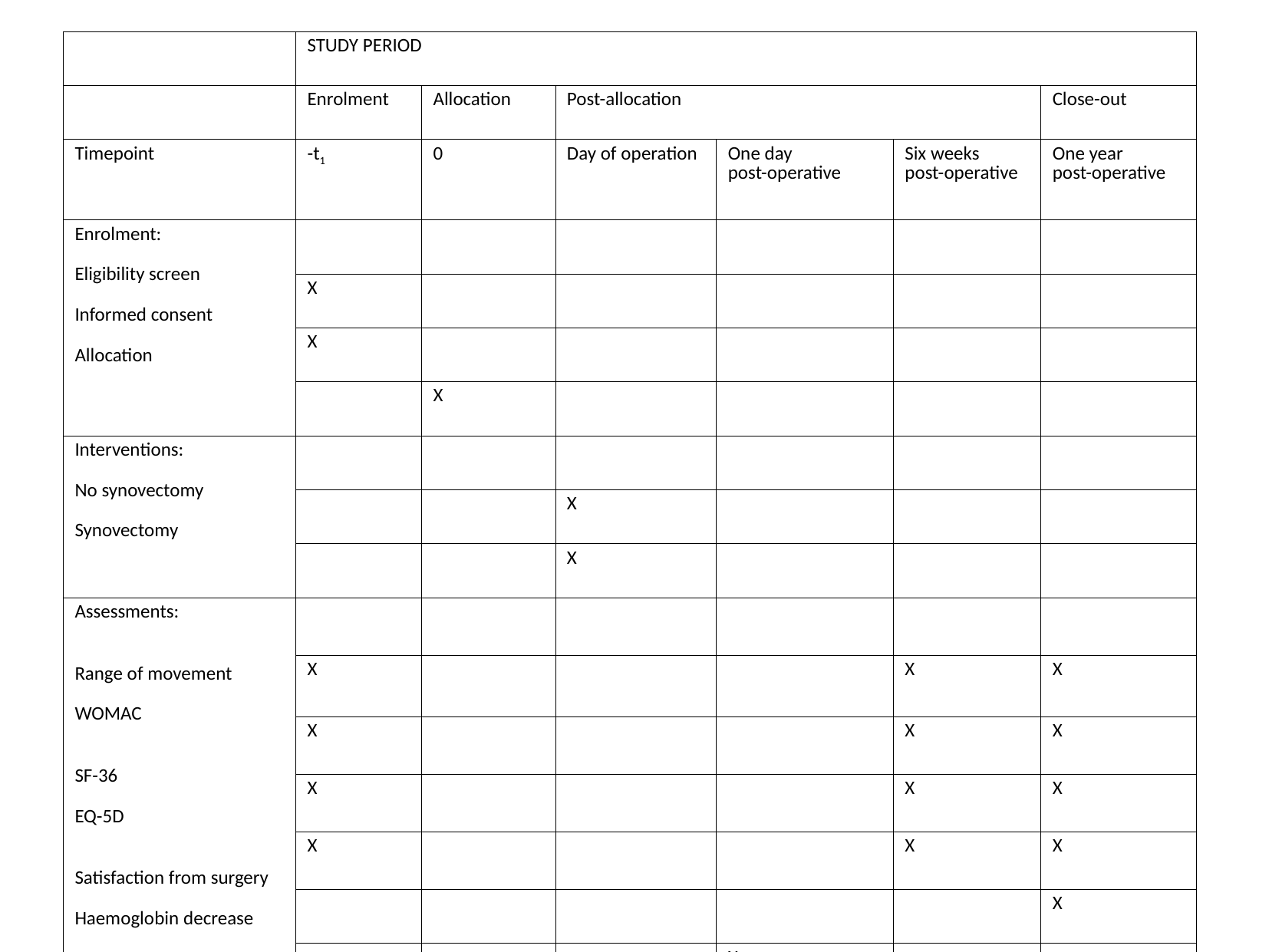

| | STUDY PERIOD | | | | | |
| --- | --- | --- | --- | --- | --- | --- |
| | Enrolment | Allocation | Post-allocation | | | Close-out |
| Timepoint | -t1 | 0 | Day of operation | One day post-operative | Six weeks post-operative | One year post-operative |
| Enrolment: Eligibility screen Informed consent Allocation | | | | | | |
| | X | | | | | |
| | X | | | | | |
| | | X | | | | |
| Interventions: No synovectomy Synovectomy | | | | | | |
| | | | X | | | |
| | | | X | | | |
| Assessments: Range of movement WOMAC SF-36 EQ-5D Satisfaction from surgery Haemoglobin decrease | | | | | | |
| | X | | | | X | X |
| | X | | | | X | X |
| | X | | | | X | X |
| | X | | | | X | X |
| | | | | | | X |
| | | | | X | | |
